# Rapid Determination of Oxygenated and Parent Polycyclic Aromatic Hydrocarbons in Milk Using Supercritical Fluid Chromatography-Mass Spectrometry

**DOI:** 10.3390/foods11243980

**Published:** 2022-12-08

**Authors:** Limin Zhang, Wei Li, Shimin Wu

**Affiliations:** Department of Food Science and Technology, School of Agriculture and Biology, Shanghai Jiao Tong University, 800 Dongchuan Road, Shanghai 200240, China

**Keywords:** supercritical carbon dioxide, pasteurization, fat content, toxic equivalency factor, dibenzopyrenes, carcinogen

## Abstract

Liquid milks are consumed worldwide in large amounts, especially by adolescents and infants. Thus, their health quality linked with polycyclic aromatic hydrocarbon (PAH) contamination has attracted great concern. This study developed a rapid and sensitive supercritical fluid chromatography (SFC)-MS method to determine two typical oxygenated PAHs (OPAHs) and EU 15+1PAHs except for benzo[k]fluoranthene (BkF) in three types of liquid milks: 10 ultra heat treated (UHT) milks, 8 pasteurized milks, and 4 extended-shelf-life pasteurized milks. The instrumental analysis was 15 min with a recovery of 67.66–118.46%, a precision of 1.45–14.68%, detection limits of 0.04–0.24 μg/kg, and quantification limits of 0.13–0.78 μg/kg. We found 9-fluorenone, anthraquinone, 15 EU priority PAHs, and benzo[a]pyrene toxic equivalent quantity (BaPeq) in the 22 milk samples, which were 0.32–1.56 μg/kg, 0.40–1.74 μg/kg, 0.57–8.48 μg/kg, and 0.01–17.42 μg/kg, respectively. The UHT milks and whole fat milks showed higher PAH concentrations than other investigated samples, where the maximum levels of BaP and PAH4 were 0.77 and 3.61 μg/kg, respectively. PAH4 dominantly contributed to the PAH8 concentration and was detected in 73% and 32% of samples at more than 1.0 and 2.0 μg/kg, respectively. The results suggest that raw milks should be strictly monitored and extensively investigated for PAH4 and BaP concentrations for future risk assessment, limitations, and dietary guidance.

## 1. Introduction

Polycyclic aromatic hydrocarbons (PAHs) and their oxygenated derivatives (OPAHs) have been shown to be carcinogenic, teratogenic, and mutagenic. They are ubiquitous and widely present in soil, water, air, sediment, dust, and food. Currently, 24 well-known parent PAHs (the US EPA 16 PAHs and EU 15+1PAHs) are of academic concern worldwide [1]. OPAHs are considered more harmful than their parent PAHs despite their undisclosed toxic mechanism.

Milk is favored by consumers worldwide due to its indispensable nutritional value. In China, nearly 400 million people eat dairy products every day. In 2020, China produced 34.4 million tons of milk, and imported dairy products were translated into approximately 19 million tons of fresh milk. According to the Chinese Nutrition Society’s recommendation of a daily 300 g milk intake, there is still a gap of nearly two-thirds of the total nutritional demand for dairy products in China. In fact, milk consumers in China have been rapidly growing. Moreover, milk is the main inseparable ingredient of many foods and beverages, including highly sensitive infant formula. Therefore, high concern must be assigned to persistent organic pollutants such as PAHs in milk and milk products. Foods of animal origin are considered the main source of dietary intake of PAHs, including milk and dairy products [1].

PAHs in milks mainly come from two routes: pollution in the process of pasture feeding [2] and generation or introduction in the process of production, processing, and transportation [3]. At present, the safety limit of benzo[a]pyrene (BaP) and PAH4 in liquid milk has not been regulated worldwide but considering that the BaP limit in drinking water in China and many developed countries is 0.01 μg/L, the content of carcinogenic PAHs such as BaP in fresh milk and sterilized liquid milk should be highly vigilant. EU has extremely strict limits on PAHs for infant formula, among which the limits of BaP and PAH4 (Benz[a]anthracene, chrysene, benzo[b]fluoranthene, BaP) are not allowed to exceed 1.0 μg/kg. Our previous study indicated that BaP in 31 commercial infant formula samples was below 1.0 μg/kg, but the PAH4 content in seven samples was 1.08–2.41 μg/kg, which exceeded the limit value of the EU [4]. It is necessary to strengthen the monitoring of BaP, PAH4, PAH8 (PAH4, benzo[k]fluoranthene, indeno-[1,2,3-cd]pyrene, dibenz[a,h]anthracene, benzo[ghi]-perylene), EU 15+1PAH (PAH8, benzo[c]fluorene, cyclopenta[cd]pyrene, 5-methylchrysene, benzo[k]fluoranthene, benzo[j]fluoranthene, dibenzo[a,l]pyrene, dibenzo[a,e]pyrene, dibenzo[a,i]pyrene, and dibenzo[a,h]pyrene) and OPAHs in raw milk and liquid milk.

The improvement of analytical methods for trace contaminants in food is endless, and the analysis of PAHs is no exception. Current quantitative analysis methods to detect PAH in milks and milk products are mostly based on chromatographic separation. The most commonly used methods are GC–MS [4,5,6,7,8,9] and HPLC-FLD [10,11,12]. SFC combines the advantages of both GC and UPLC and is compatible with various types of detectors. The combination of SFC and MS greatly improves the sensitivity and efficiency of analysis. SFC-MS uses supercritical CO_2_ as the mobile phase, which effectively reduces toxic and expensive organic solvents. Supercritical CO_2_ and n-hexane have similar polarities, which is suitable for the analysis of weakly polar substances such as PAHs [13]. Supercritical CO_2_ is significantly less viscous than liquid, and the diffusivity of analyte is much higher than that in liquid [14]. Thus, SFC analysis can be performed at a high flow rate without increasing the pressure, which significantly shortens the analysis time. Increasing attention has been paid to SFC due to the solvent reduction characteristic, environmentally friendly nature, and green chemistry requirements [15,16].

Compared with traditional HPLC and GC, SFC exhibits faster and better separation ability. For example, the quantification of EU 15+1PAHs in coffee and dark beer can be completed within only 14 min using SFC-MS with detection limits below 1 μg/kg [16]. Another example reported that SFC-MS enabled rapid online monitoring of EPA PAH16 in particulate matter samples using an online supercritical fluid extraction-MS detection system [17].

The purpose of this study was to develop a rapid and sensitive analytical method to determine EU 15+1PAHs and OPAHs in milk by using SFC-QqQ-MS. The method was optimized and validated to investigate the PAH concentrations of 22 real milk samples, including 10 UHT milks, 8 pasteurized milks, and 4 extended-shelf-life pasteurized milks. The influencing factors of PAHs in different types of milks were also discussed.

## 2. Materials and Methods

### 2.1. Reagents and Samples

All organic solvents in this study were of HPLC grade. Acetonitrile, acetone, n-hexane, and methanol were supplied by CNW Technologies (Dusseldorf, Germany). Deionized water was purified using a Milli-Q water purification system (Millipore, MA, USA). The mixed EU 15+1PAH standard cyclohexane solution (>99.8%) was ordered from ANPEL Laboratory Technologies (Shanghai, China). It consists of benzo[c]fluorene (BcF), benz[a]anthracene (BaA), cyclopenta[cd]pyrene (CP), chrysene (Chr), 5-methylchrysene (5-MChr), benzo[b]fluoranthene (BbF), benzo[k]fluoranthene (BkF), benzo[j]fluoranthene (BjF), benzo[a]pyrene (BaP), indeno-[1,2,3-cd]pyrene (IP), dibenz[a,h]anthracene (DahA), benzo[ghi]-perylene (BghiP), dibenzo[a,l]pyrene (DalP), dibenzo[a,e]pyrene (DaeP), dibenzo[a,i]pyrene (DaiP), and dibenzo[a,h]pyrene (DahP). Standard cyclohexane solutions (>99.8%) of two OPAHs, i.e., 9-fluorenone (9-FO) and anthraquinone (ATQ), were purchased from Dr. Ehrenstorfer GmbH (Augsburg, Germany). All standard solutions were diluted to 1 mg/mL using n-hexane and subsequently stored at −20 °C in darkness.

The QuEChERS extraction tube, EMR-Lipid tube, and polish tube were obtained from Agilent Technologies (Santa Clara, CA, USA). The extraction tube included 6 g magnesium of sulfate and 1.5 g of sodium acetate. The EMR-Lipid tube included 1 g of adsorbents (compositions unknown), and the polish tube included 1.6 g of magnesium sulfate and 0.4 g of sodium chloride.

Twenty-two commercial milk samples were collected from local supermarkets in Shanghai, China. The milk samples were commonly consumed, including two from Germany and one from Australia. They consisted of 10 UHT milks (M1–10), 8 pasteurized milks (M11–18), and 4 extended-shelf-life pasteurized milks (M19–22) with different fat contents. The UHT milks were stored at room temperature, and the other two types of milk were stored at 4 °C until analysis.

### 2.2. Sample Pretreatment

The procedures of PAH extraction and cleanup were conducted according to our previous studies [4,5]. In brief, milk samples were extracted by a mixture of acetonitrile/acetone (2:1, *v/v*) and subsequently successively purified by a QuEChERS extraction salt pack, EMR-Lipid tube, and polish tube. Finally, the extract was concentrated using a nitrogen stream to approximately 20 mg and reconstituted using n-hexane to 200 μL.

### 2.3. SFC-QqQ-MS Analysis

Separation: A Waters Acquity UPC2 instrument (Milford, MA, USA) was used to separate the n-hexane extract. The SFC separation column was ACQUITY HSS C18 SB (3.0 × 100 mm, 1.8 μm) supplied by Waters (MA, USA), and its temperature was held at 40 °C. The injection volume was 5 μL. Solvents A (CO_2_) and B (methanol) were selected as mobile phases, and the gradient elution was as follows: 1% B (0–0.5 min), 10% B (8 min), 20% B (10.5 min), 35% B (11 min), 50% B (11.1–12.5 min), and 1% B (13–15 min). The flow rates were set to the gradient mode as follows: 1.5 mL/min (0–11 min), 1.0 mL/min (11.1–13 min), and 1.5 mL/min (13.5–15 min). The back-pressure was 1600 psi.

Identification: A Xevo TQ-XS (Waters, MA, USA) was linked to UPC2 to identify MS. Methanol with 0.1% formic acid was used as the make-up solvent for MS at a flow rate of 0.2 mL/min. The APCI ion source was used to detect MS in the positive mode. The probe temperature was set at 450 °C, and the desolvation gas flow rate was 1000 L/h. The mass detector was operated in the selected-ion monitoring (SIM) mode with a corona pin voltage value of 2.0 kV. The SIM conditions are shown in Table 1.

### 2.4. Method Validation

Six UHT milk samples with low concentrations of PAHs were spiked in two levels of 5.0 and 10.0 μg/kg for recovery and precision tests. The calculation of recovery has deducted the concentrations of PAHs in the pseudo blank samples. PAH standard solutions at levels of 1, 2, 5, 10, 20, 40, 60, and 100 μg/kg were tested for linearity. The limits of determination (LODs) were determined using S/N = 3, and the limits of quantification (LOQs) were calculated using S/N = 10. The LODs and LOQs were estimated with 10 blank milk samples and spiked samples (0.5 μg/kg), and PAH calibration curves at 0.2, 0.4, 0.6, 0.8, and 1.0 μg/kg were used.

### 2.5. Statistical Analysis

The analysis of the sample was conducted in triplicate (*n* = 3). The statistical calculations were performed by SPSS 24.0 software.

## 3. Results and Discussion

### 3.1. Optimization of the Conditions of SFC Separation and MS Ionization

By using an HSS C18 SB column, the separation and elution of two OPAHs and EU 15+1PAHs could be completed within 11 min. Since the separation of benzofluoranthenes (BbF, BkF, and BjF) and dibenzopyrenes (DaeP, DaiP, and DahP) was not favorable, further optimization was conducted to obtain the ideal separation parameters.

**Figure 1:**
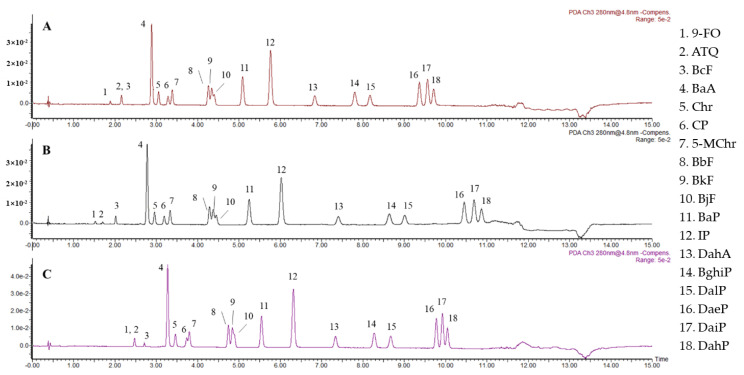
SFC chromatography of 18 PAHs in different mobile phases (1000 μg/kg). (**A**) Methanol/acetonitrile (1:1, *v/v*), (**B**) methanol, and (**C**) acetonitrile (0.5% formic acid), and 1–18 peaks represent 9-FO, ATQ, BcF, BaA, Chr, CP, 5-MChr, BbF, BkF, BjF, BaP, IP, DahA, BghiP, DalP, DaeP, DaiP, and DahP, respectively.

Usually, a mixture of CO_2_ and organic cosolvent is used as the mobile phase to improve the solubility of analytes and avoid precipitation during the injection. During the elution process, organic cosolvents are one of the key factors that affect the peak shape and retention time. Therefore, selecting an appropriate cosolvent is necessary for satisfactory separation [18]. In this study, the composition of the mobile phase was optimized with three different organic cosolvents: methanol, acetonitrile, and formic acid. To compare the separation ability of mobile phases with different polarities and pH values, methanol, methanol/acetonitrile (1:1, *v/v*), and acetonitrile (0.5% formic acid) were selected as mobile phase B. The retention time varied with the change in polarity and pH. When methanol with a strong polarity was used, the resolution of PAHs was good except for BbF, BkF, and BjF. When methanol/acetonitrile (1:1, *v/v*) with a weaker polarity was used, the solubility of PAHs increased with decreasing polarity. The total elution time was shortened by approximately 1 min, but the resolutions of the two OPAHs and two groups of isomers (BaA and Chr, DaeP and DaiP and DahP) were poorer (Figure 1). When acetonitrile (0.5% formic acid) was used, the separation of the two groups of isomers deteriorated, which indicates that the reduction in pH was not conducive to the separation of PAHs. Thus, methanol was selected as mobile phase B.

After the mobile phase was determined, the flow rate and column temperature were chosen at 1.5 mL/min and 40 °C, respectively. During the condition adjustment, we observed that the resolution between the isomers slightly decreased after the initial flow rate decreased from 1.5 mL/min to 1.2 mL/min. The reason may be that the decrease in the flow rate widened the peaks and resulted in a decrease in resolution. We also found that the separation of dibenzopyrenes at 40 °C was better than that at 50 °C because high-molecular-weight PAHs were less soluble in supercritical CO_2_ at higher temperatures [19].

Finally, the MS response was optimized. With increasing corona pin voltage (2.0–2.5 kv), the MS responses of ATQ, BcF, and BaP were stronger, but the responses of other analytes decreased. The responses of BcF improved if the corona pin voltage continued to increase. However, the responses of the other 14 PAHs (except for ATQ and BaP) were reduced. Thus, we chose 2 kV as the corona pin voltage, which also ensured strong intensities of ATQ and BaP. Methanol with different proportions of formic acid was used as the make-up solvent to improve ionization by adjusting the pH. When the composition of formic acid in methanol increased from 0.1% to 0.2%, the response of PAHs decreased, which indicates that the reduction of pH was not conducive to ionization. Similarly, when the flow rate increased from 0.2 mL/min to 0.4 mL/min, the response of each PAH decreased. Therefore, methanol containing 0.1% formic acid was selected as the make-up solvent, and the flow rate was set at 0.2 mL/min.

**Figure 2:**
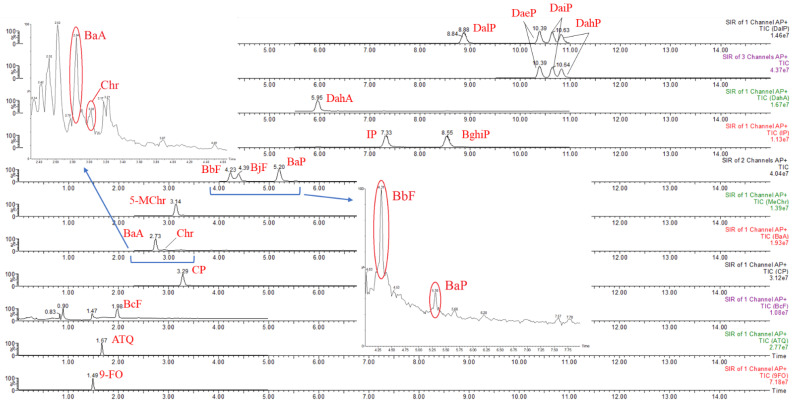
Optimized SFC-QqQ-MS chromatograms of 17 PAHs standards (200 μg/kg) and PAH4 in real milk samples.

After the above-mentioned optimization, an acceptable chromatographic separation of the 17 PAHs was achieved (Figure 2). Except for three benzofluoranthenes (BbF, BkF, and BjF), the separation of other neighboring PAHs is satisfactory with resolution values greater than 1.0, which indicates that most of the PAH peaks can be well separated. In comparison, the resolution values of BbF/BkF and BkF/BjF were 0.82 and 0.73, respectively. The quantitative ions of these three isomers are identical (m/z = 253), so their unsatisfactory resolution will inevitably affect the quantitative analysis. Nevertheless, the influence of the poor separation of BkF on its quantitation can be ignored due to its trace lack of detection in milk samples [4] and weak response in this method. The quantitation of BbF and BjF can be well determined since their resolution is above 1.0. If necessary, special columns and mobile phases can be explored for the BkF well separation from its two isomers (BbF and BjF) when using SFC-QqQ-MS for a synchronous quantitative analysis of EU 15+1PAHs.

### 3.2. Method Validation

The results are shown in Table 2. All analytes had satisfactory linearity with the R^2^ value > 0.99 at the concentration range of 1.0−100.0 μg/kg. At the 5-μg/kg spike level, the recoveries of 17 target compounds were 67.66–118.46%. This recovery conformed to the requirements of EU regulations (EU No 836/2011) on trace analyte quantification. The calculated LOD and LOQ values were 0.05–0.28 μg/kg and 0.13–0.73 μg/kg, respectively. The LOQs of three dibenzopyrenes (DaeP, DaiP, and DahP) significantly improved compared with our GC–MS method [4]. This result is mainly attributed to the better dissolution ability of supercritical CO_2_ for PAHs with a high molecular weight and high boiling point. Thus, the chromatographic response of the four dibenzopyrenes was enhanced. This is of great significance for the quantification of PAHs that contain six or more benzene rings in foods, water, beverages, and drinks with ppb to trace PAH levels. The only disadvantage of this method compared to GC–MS is that BkF cannot be determined. This is due to the poor chromatographic separation of BkF with a weak response covered by adjacent peaks in the SFC chromatogram. For milk, it is difficult to achieve accurate quantification of BkF in real samples considering its trace occurrence based on our previous investigation [4]. Therefore, the following application analysis on quantification of PAHs in milk samples targeted only the two OPAHs and EU 15+1PAHs except for BkF.

### 3.3. Application of the Developed Method for Real Sample Analysis

Twenty-two commercial milk samples were successfully analyzed using the established SFC-QqQ-MS method. Table 3 shows the PAH4 and PAH8 concentrations and the BaP toxic equivalent quantity (BaPeq) of the 22 samples [2]. Because there were no reliable toxic equivalency factor (TEF) data, the calculation of BaPeq did not contain BcF, 9-FO, or ATQ. The results indicate that the total concentrations of 17 PAHs in the 22 tested samples were 1.63–11.01 μg/kg. The average PAH4 concentrations in the UHT milks, pasteurized milks, and extended-shelf-life pasteurized milks were 2.27 μg/kg, 1.08 μg/kg, and 1.28 μg/kg, respectively. Seven samples contained more than 2.0 μg/kg PAH4, which was approximately 32% of the investigated products. As a PAH4, BaP was present at concentrations of 0.41–0.77 μg/kg in 9 UHT samples, which consisted of a 90% BaP rate in all tested UHT products. In contrast, BaP was not detected in pasteurized milks or extended-shelf-life pasteurized milks. The BaP concentrations in all samples were lower than 1.0 μg/kg, while the PAH4 concentrations in 73% of the samples were higher than 1.0 μg/kg.

This result suggests that raw milks should be strictly monitored for PAH4 and BaP concentrations if the infant formula considers the legal limitations of BaP and PAH4 by the EU (<1.0 μg/kg). Theoretically, according to the conversion rate of fresh milk into milk powder, fresh milk containing both BaP and PAH4 should not exceed 0.14 μg/kg [4]. In this view, all 22 samples were not suitable for infant formula production, since their PAH4 concentrations were much more than 0.14 μg/kg. Three other PAHs (Chr, BaA, and BbF) showed detection rates of 86%, 59%, and 50%, respectively.

DahA was detected in two samples, so its health risk should be highly considered due to its TEF at 5.0. Four dibenzopyrenes (DalP, DaeP, DaiP, and DahP) were detected in seven samples. The detection rates of DalP, DaeP, DaiP, and DahP were 27%, 14%, 14%, and 5%, respectively. Although these four analytes had generally low concentrations, their TEFs were relatively high. DalP belongs to class-2A carcinogens, while DaeP, DaiP, and DahP belong to class-3 and -2B carcinogens, respectively. DaeP had a TEF of 1.0, but all TEFs of DalP, DaiP, and DahP were 10.0, which increased BaPeq of the samples where dibenzopyrenes were detected. For the two samples with BaPeq above 10.0, the contributions of dibenzopyrenes were 71% and 92%. In fact, dibenzopyrenes were the main contributors of BaPeq in each sample, which is consistent with the results of environmental samples [20]. In particular, these four analytes were simultaneously detected in one milk sample, and three of them were simultaneously detected in the other two milk samples.

The detection rate of the two OPAHs was 100%, which is consistent with our previous results [4]. The parent PAH of 9-FO is fluorene, and the parent PAH of ATQ is anthracene, both of which belong to the US EPA 16PAHs. Our previous study investigated 89 milk samples from 9 different countries, which indicates a much higher average concentration of anthracene than that of other PAHs [5]. The high content of anthracene may cause a high concentration of ATQ.

### 3.4. Influencing Factors of PAH Content in Different Types of Milks

Pasteurized milks are commonly considered better in flavor and nutrition, but they are limited by shorter shelf life (seven days) and lower storage temperature than UHT milks. Currently, the dominant liquid milk products in the Chinese market are UHT milks. In general, UHT milks enjoy six to twelve months of shelf life. Alternatively, a new product named extended shelf life milks appeared in recent years [21]. They combined pasteurization treatment with new processing technology to extend their shelf life to 14–20 days and can be considered a special type of pasteurized milk that also must be stored at 4 °C.

**Figure 3:**
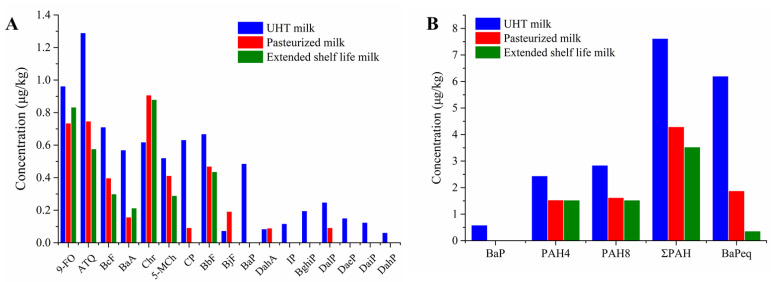
PAH concentrations in milks with different sterilization processes. (**A**) individual PAH analytes. (**B**) Concentrations of BaP, PAH4, PAH8, 17 PAHs, and BaPeq.

Figure 3A shows the average concentrations of 17 PAHs in different types of milks. The top concentrations of the three PAHs were ATQ, 9-FO, and BcF in UHT milk; Chr, ATQ, and 9-FO in pasteurized milk; and Chr, 9-FO, and ATQ in extended-shelf-life pasteurized milk. Two OPAHs were detected in all tested samples, and no significant difference was observed in the concentrations of different types of milks. In pasteurized and extended-shelf-life pasteurized milks, the Chr concentration was higher than that in UHT milks; the BaA, CP, and BbF contents were significantly lower than those in UHT milk (*p* < 0.05); and the BaP, BghiP, DaeP, DaiP, and DahP concentrations were all below the LOQ. In previous studies, pasteurized milk also had significantly higher Chr concentrations and significantly lower BaA and IP concentrations than UHT milk [5,12]. In addition, Naccari et al. [3] found that unheated raw milk had significantly lower PAH concentrations than pasteurized or UHT-treated milk. The reason is that PAHs in raw milk only came from the environment, while PAHs in pasteurized milk were the sum of PAHs from the environment and the heating process. Rawash et al. [22] also found that raw milk had lower PAH concentrations than pasteurized milk and UHT milk in the detection of PAH pollution in Egyptian milk. Overall, these results confirm that the factory sterilization processing of milk may form PAHs and OPAHs. How to minimize the influence of processing on PAHs and OPAHs in milks requires further study.

**Figure 4:**
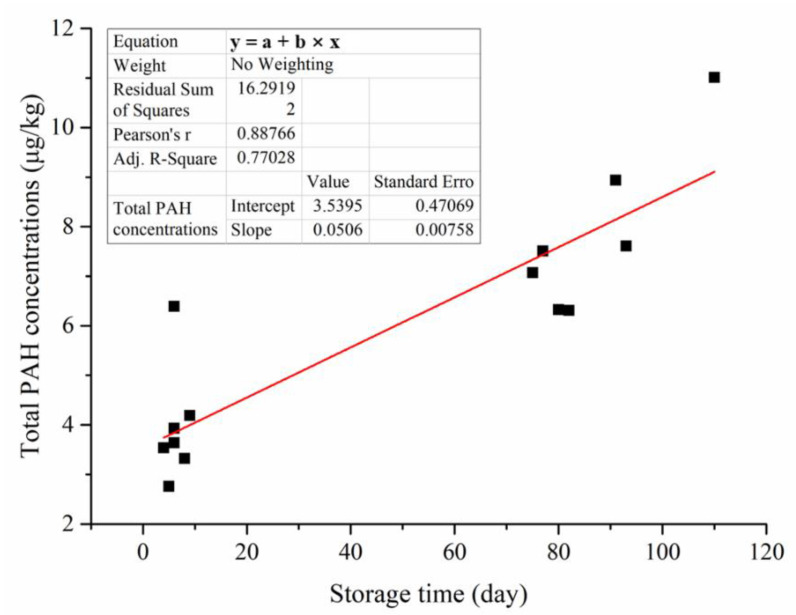
Correlation analysis of storage time and total PAH concentration in milks. Red line: the linear regression model.

Figure 3B shows the difference in average PAH concentrations in three different types of milks. UHT milks had a higher total PAH concentration than pasteurized milk and extended-shelf-life pasteurized milk. In this study, the detection rates of heavy PAHs in pasteurized milks were lower than those in UHT milks. Therefore, the total PAH concentration and BaPeq of UHT milks were significantly higher. In addition to the influence of sterilization temperature and time, different storage times may affect PAHs and OPAHs in the milk samples. A previous study found that the concentrations of PAHs in soybean oil and rapeseed oil significantly increased with storage time, and the concentrations increased faster at room temperature than at 4 °C [23]. Pasteurized milk and extended-shelf-life pasteurized milks had a shelf life of less than 20 days and were stored at 4 °C, while UHT milks had a shelf life of at least six months and were stored at room temperature. During storage, oxidation, migration, and transformation of PAHs occur in milks, which changed the PAH and OPAH concentrations. Overall, the extended-shelf-life pasteurized milks had lower total PAH concentrations than pasteurized milks. Based on the total PAH concentration and storage time data of 14 whole milks, we calculated the Pearson correlation coefficient between storage time and total PAH concentrations and obtained 0.89 (Figure 4). This result suggests that the concentration of PAHs in milks increased with storage time.

**Figure 5:**
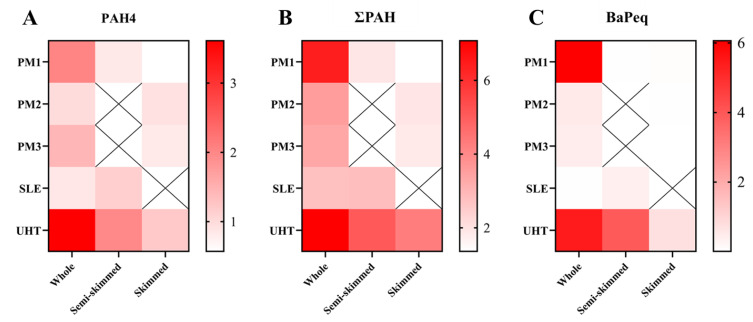
Comparison of PAHs among milk samples with different fat contents. PM: pasteurized milk, ESL: extend-shelf-life pasteurized milks. (**A**) concentration of PAH4 (BaP, BaA, Chr, BbF), (**B**) Concentration of 17 PAHs, (**C**) BaPeq of 17 PAHs.

Figure 5 compares the concentrations of PAH4, total PAH, and BaPeq in milk samples with different fat contents. In the pasteurized milks and UHT milks, the concentrations of PAHs in whole milks were higher than those in the corresponding skim and semi-skim samples. Various studies have proven that the concentration of PAHs in milks is positively correlated with fat content [4,24,25]. This study calculated the BaPeq value between whole milks and semi-skim/skim milks, which indicates significant differences between whole milks and semi-skim/skim milks. Thus, more attention should be given to the potential PAH health risks of whole milks.

## 4. Conclusions

This study established an SFC-QqQ-MS method with the advantage of rapid, sensitive, and solvent-saving nature to determine PAH and OPAH in milks. The EU 15+1PAHs except for BkF and two OPAHs (9-FO and ATQ) were well separated and determined in 22 different commercial milks. Compared with GC–MS, the instrument analysis time was shortened by 1/2, and the LOQs of four dibenzopyrenes (DalP, DaeP, DaiP, and DahP) were significantly lower. Higher PAH concentrations were detected in the UHT milks compared to pasteurized milks and whole milks compared to semi-skim/skim milks. This result suggests that more attention should be given to the potential PAH health risks of UHT milks and whole milks. The noticeable BaPeq in the 22 milk samples indicates that the maximum liquid milk intake should be defined for populations of different ages. Further studies are necessary to explain the lower PAH level in extended-shelf-life pasteurized milks, elucidate the occurrence of dibenzopyrenes in milks, and minimize the influence of processing on PAHs and OPAHs in milks, including sterilization, skimming, and storage conditions.

## Figures and Tables

**Table 1:** SIM parameters of target analytes using UPCC-QqQ-MS.

PAHs	Retention Time (min)	Quantification Ion (m/z)	Dwell Time (ms)	Scan Start Time (min)	Corn Voltage (V)
9-FO	1.55	181	100	0.5	20
ATQ	1.73	209	100	0.5	15
BcF	2.02	217	100	1.0	50
BaA	2.77	227	100	2.0	40
Chr	2.94	227	100	2.5	10
5-MChr	3.17	243	100	2.5	10
CP	3.31	229	100	2.5	20
BbF	4.26	253	100	4.0	40
BjF	4.40	253	100	4.0	60
BaP	5.21	253	100	4.5	20
DahA	5.96	279	200	5.5	35
IP	7.31	279	200	7.0	35
BghiP	8.52	277	200	7.5	30
DalP	8.86	303	200	8.0	25
DaeP	10.38	303	200	9.5	40
DaiP	10.65	303	100	9.5	40
DahP	10.82	303	100	9.5	50

**Table 2:** Validation results of the established UPCC-QqQ-MS method.

PAHs	R^2^	Low Spike Level 5 μg/kg (%)			Low Spike Level 10 μg/kg (%)			LOD (μg/kg)	LOQ (μg/kg)
		Recovery	RSD_r_^a^	RSD_R_^b^	Recovery	RSD_r_^a^	RSD_R_^b^		
9-FO	0.9974	67.66	8.94	12.93	72.46	6.50	6.19	0.04	0.13
ATQ	0.9948	87.77	11.44	13.25	76.80	4.19	8.33	0.11	0.37
BcF	0.9914	118.00	10.23	13.60	106.00	3.23	8.84	0.16	0.53
BaA	0.9935	100.46	3.39	10.89	108.11	12.05	12.70	0.06	0.20
Chr	0.9967	81.83	0.65	6.03	78.40	1.45	5.34	0.18	0.60
5-MChr	0.9969	103.54	1.78	4.72	99.66	4.41	8.65	0.10	0.32
CP	0.9948	110.00	0.32	4.52	93.09	4.59	5.31	0.12	0.40
BbF	0.9963	118.46	8.73	14.59	86.29	4.38	5.86	0.22	0.73
BjF	0.9964	112.23	3.72	8.41	94.97	7.00	11.69	0.24	0.78
BaP	0.9941	114.74	2.67	5.86	112.23	4.47	4.81	0.10	0.33
DahA	0.9919	108.91	5.98	8.42	103.66	6.20	10.87	0.12	0.38
IP	0.9963	109.83	4.88	8.24	110.51	6.05	14.68	0.12	0.41
BghiP	0.9967	109.03	7.14	9.05	98.51	4.25	8.55	0.13	0.42
DalP	0.9965	70.06	4.36	5.90	73.71	3.84	5.19	0.13	0.43
DaeP	0.9932	88.46	4.85	6.83	86.29	4.65	6.79	0.10	0.34
DaiP	0.9940	93.60	6.95	9.56	87.77	5.16	7.98	0.08	0.25
DahP	0.9933	90.29	3.56	6.14	107.66	4.09	6.36	0.15	0.51

^a^ The relative standard deviation of repeatability, *n* = 6. ^b^ The relative standard deviation of intermediate precision, *n* = 6.

**Table 3:** OPAH and PAH concentrations of 22 commercial milk samples by UPCC- QqQ-MS.

Samples	Concentration (μg/kg)						
	OPAHs		Parent PAHs				
	9-FO	ATQ	BaP	PAH4	PAH8	EU PAHs ^a^	BaPeq ^b^
M1	0.91	1.62	0.41	2.01	3.51	8.48	17.42
M2	0.59	1.74	0.50	1.44	2.05	5.28	12.18
M3	1.15	1.10	0.47	2.80	3.30	6.69	6.44
M4	0.78	1.13	0.49	2.37	2.37	4.42	1.66
M5	1.56	0.87	0.46	3.61	3.61	4.64	5.40
M6	0.88	1.52	0.45	3.28	3.28	5.41	4.30
M7	1.15	1.60	0.54	1.66	1.66	3.92	7.90
M8	0.74	1.29	Nd ^c^	1.98	1.98	3.03	3.91
M9	0.82	1.03	0.66	1.21	1.21	2.42	0.72
M10	0.68	0.74	0.77	2.38	2.96	4.77	0.94
M11	0.96	0.56	nd ^c^	1.43	1.43	1.80	0.44
M12	0.43	1.27	nd ^c^	2.02	2.38	4.69	6.06
M13	1.09	0.56	nd ^c^	0.98	0.98	1.89	0.53
M14	0.39	0.40	nd ^c^	0.57	0.57	0.57	0.57
M15	0.50	0.51	nd ^c^	0.83	0.83	0.83	0.01
M16	0.44	0.63	nd ^c^	0.84	0.84	0.84	0.01
M17	0.46	0.60	nd ^c^	1.02	1.02	2.87	0.49
M18	0.45	0.56	nd ^c^	0.92	0.92	0.92	0.01
M19	0.77	0.66	nd ^c^	1.86	1.86	2.21	0.46
M20	0.32	0.56	nd ^c^	1.14	1.14	1.92	0.37
M21	0.87	0.58	nd ^c^	0.85	0.85	1.31	0.01
M22	0.86	0.49	nd ^c^	1.26	1.26	2.84	0.62

^a^ EU 15+1 PAHs except for BkF. ^b^ BaPeq was calculated using the 15 parent PAHs. ^c^ nd: not detected.

## Data Availability

No data was used for the research described in the article.

## Abbreviations

ATQ	anthraquinone
BaA	benz[a]anthracene
BaP	benzo[a]pyrene
BbF	benzo[b]fluoranthene
BcF	benzo[c]fluorene
BghiP	benzo[ghi]perylene
BjF	benzo[j]fluoranthene
BkF	benzo[k]fluoranthene
Chr	chrysene
CP	cyclopenta[cd]pyrene
DaeP	dibenzo[a,e]pyrene
DahA	dibenz[a,h]anthracene
DahP	dibenzo[a,h]pyrene
DaiP	dibenzo[a,i]pyrene
DalP	dibenzo[a,l]pyrene
9-FO	9-fluorenone
IP	indeno [1,2,3-cd]pyrene
5-MChr	5-methylchrysene
QuEChERS	quick, easy, cheap, effective, rugged, and safe
